# American Academy of Dental Science

**Published:** 1902-08

**Authors:** 

**Affiliations:** American Academy of Dental Science


### AMERICAN ACADEMY OF DENTAL SCIENCE.

The regular monthly meeting of the American Academy of Dental Science was held at Young's Hotel, Boston, on Wednesday evening, February 5, 1902, at 6 o'clock, President Bradley in the chair.

Two papers were read, the first by Thomas Fillebrown, M.D., D.M.D., entitled "Physiological Results of Operation for Cleft Palate," with cases; the second by Eugene H. Smith, D.M.D., entitled "A Contribution to Operative Orthodontia," illustrated with stereopticon.

*President Bradley.*—I have the pleasure of calling the attention of the Fellows of the Academy to this article which I hold in my hand, a piece of carving done by Dr. Belyea, with the help of the dental engine. It is a fine specimen of artistic work, and I think the Fellows of the Academy will be glad to see it. I will pass it around.

*Dr. Belyea.*—It is done on steel, and it is not quite finished.

There is a good deal of work to be done on it before I should want to say it was finished.

*President Bradley.*—This evening we have quite a programme to present to the Academy, but at this time I will say that if any Fellow has an incident of office practice to present, instruments, or anything of that kind, now is an opportunity.

If there is nothing of that character to be presented, before we proceed to the consideration of the papers Dr. Cecil P. Wilson has a matter which he would like to bring before the Academy at this time.

*Dr. Wilson.*—About two months ago those of us who are members of the Dental Protective Association received a circular from Dr. Crouse, announcing his intention and desire to give up the labors which he has carried on his own shoulders, singly and alone, for so many years. I do not know that many of the members of the Academy realize what Dr. Crouse has done for the profession. If the true story were told, it would show that many years have been devoted to the cause of our profession, at the cost to him of a large amount of valuable time and money. During the time when the Crown Company was most actively engaged, and, in fact, many years before that, Dr. Crouse gave up weeks and weeks of his time, for which he has never received a single cent, or the slightest remuneration in any way; and added to this, there is not the slightest doubt about his absolute honesty and integrity.

After consulting with some of the members of the Academy, I wrote and asked him if it would be agreeable to him if the matter were brought up before the Academy, and he said it would. I received a long communication from him, which I should like to read to-night, but think perhaps it will be better to reserve until a later meeting.

I would like to make a motion that a committee of five be appointed by the chair to take into consideration the advisability of making some substantial recognition to Dr. Crouse for his untiring labor and devotion in behalf of our profession, also to assist him to place the Dental Protective Association upon a permanent basis, the committee to have power to enlarge their number at their discretion.

*Dr. Meriam.*—I saw something of Dr. Crouse at the time this Association was formed, and I wish to second that motion. I do not think that many of us know how much Dr. Crouse has done, or what he has had to face. I can remember, and probably the memories of many of us go back to rubber days. I remember once in Salem a woman came to one of the offices, and had an impression taken, and the dentist in the evening when making a call saw an impression on the desk of one of his neighbors, who said, "I took it this morning for a woman." "We are stuck!" exclaimed dentist No. 1. She had been from office to office, and it was the business of that woman to go over the country and have teeth made, and then dentists were brought into court on the evidence obtained.

Perhaps that could not have been so in bridge-work, but it was similar. I have heard of a man giving clinics at a meeting, under contract to report to the Tooth Crown Company all the men who were doing bridge-work at the time. That sort of thing would have multiplied all over the country had not Dr. Crouse taken the stand and done the work he has. No one could have been secure. There could have been no peace in practising with spies about. Only such work as Dr. Crouse has done has made quiet practice possible. Of course, it is easy to praise Dr. Crouse, but that is not necessary, as his work has been before us.

*Dr. Fillebrown.*—I want to say, in regard to the remark that has been made here that Dr. Crouse has never received a penny, that I have been on a committee several times that has looked over these books and audited them, and I am very sure that he has never taken a penny from the treasury. The idea of this, as I understand it, is to start a movement that shall result in some substantial return to Dr. Crouse for his many sacrifices and long service, and not simply from the Academy, but that it shall be country-wide. I did not hear the resolution, but I understood it was to act in conjunction with other committees.

*Dr. Wilson.*—I intended to leave that entirely with the committee.

*Dr. Fillebrown.*—My idea is that the committee should take into consideration not simply what the Academy will do, but stimulate other societies to do something. This, I understand, is done not without the knowledge of some New York men already, and it may extend to other societies and be a national movement.

*Dr. Brackett.*—If I rightly understand the reference to the circular that Dr. Crouse sent out, there was some expression in it to the effect that he had carried this burden without compensation, and that he would be under the necessity of withdrawing, looking forward to future years and to making provision for his family. I immediately wrote Dr. Crouse, in season to be presented at the annual meeting of the members of the Protective Association, that I felt that his services should be retained in behalf of the Protective Association, and that they should be amply compensated; that there were other men who could fill teeth, other men who could carry on the routine of dentistry. I question if there is another man who can do that particular work as he has done it.

I sincerely feel that this movement should be something more than an expression of sympathy. It should give something in the way of substantial compensation for the time and labor and effort which he has faithfully devoted through these many years. It is my earnest hope that such action will be taken as will justify Dr. Crouse in giving to continued service in this most excellent cause, in behalf of all of us, such portion of his time as is needed, and that he will be well compensated therefor. It is the truest economy for all of us to pay Dr. Crouse for doing this work.

*Dr. Clapp.*—I want to make one suggestion that has just come into my mind. All of us would be perfectly willing to pay another assessment, as he calls it, of ten dollars to the Protective Association to protect us for some time to come. Now, if it was understood that it was rather intended that every member of this Protective Association should make a contribution of ten dollars, and let it go to Dr. Crouse, it would help him and help us.

*Dr. Dowsley.*—I have not seen the circular that has been referred to. Dr. Crouse did not send me one, but I have been in constant communication with him for two years or more on the subject of the Dental Protective Association. Last October I met him in Springfield, and I feel that I can say that I know what Dr. Crouse's desire is. I do not think that he intends to retire from the presidency of the Dental Protective Association; but he realizes that he is growing old, that he may die at any moment, that there is no one to take his place, that the membership of the Dental Protective Association is not growing as it ought, and he wants some means devised whereby the membership of the Protective Association will be increased. What that method is he would like the various associations throughout the country to suggest, and he wants to get the best. It seems to me that ten dollars from an association of this kind would be of very little help to him. The Protective Association is Dr. Crouse's monument, and we want to maintain it, and that is his idea. Dr. Crouse wishes a committee of counsellors to assist him in the management of the Association at the present time; and hereafter when he is not at the head, perhaps, they will be familiar with the workings of the Association.

*President Bradley.*—I was under the impression that Dr. Clapp's suggestion was ten dollars from each member, and not that amount from the Academy merely.

*Dr. Clapp.*—That is correct.

*Dr. Ainsworth.*—I would like to ask if there is any one present who has any idea of the amount of money we have in the Dental Protective treasury.

*Dr. Dowsley.*—I do not know the amount, but Dr. Crouse says that he has ample funds to carry on any prosecution that is coming on at the present time.

*Dr. Fillebrown.*—I presume every member here is aware of the fate of that last law-suit. It was nolle prossed on account of collusion.

*President Bradley.*—Gentlemen, we have this evening two papers to present, the first one entitled "Physiological Results of Operation for Cleft Palate," with cases, by Thomas Fillebrown, M.D., D.M.D.

(For Dr. Fillebrown's paper, see page 557.)

*President Bradley.*—I will now name the members of the Committee to consider the motion of Dr. Wilson: Dr. Wilson, Dr. R. R. Andrews, Dr. E. H. Smith, Dr. Charles A. Brackett, and Dr. Thomas Fillebrown.

DISCUSSION.

*President Bradley.*—The subject is open for discussion. I am sure the Fellows of the Academy have been pleased with the exhibition that has been made in their presence this evening, and which I hope will be prolific of discussion. We shall be glad to hear from those who wish to say something on this subject; we want the time to be utilized.

*Dr. Andrews.*—I want to express my pleasure and gratification at the demonstration which has been given here to-night. We ought to be proud of a man who can accomplish such results, proud that he is a dentist, and proud that he is one of us. I have been very much gratified. I did not suppose it was possible in so short a time to bring about such results, apparently perfect.

*Dr. Meriam.*—I remember some years ago Dr. Fillebrown brought a matter of this kind before the Harvard Odontological Society, and I said at that time that we had had dentists who had become surgeons, but they had left their dentistry behind and had adopted the old surgery,—that is, they became surgeons minus the dentistry. And now this is dentistry plus the surgery. That I said some ten or fifteen years ago, and I should echo it to-day. I think it shows what a dental training, added to an ordinary surgical training, can bring about, and I do not think it could be brought about in any other way than by the special work of the dentist being added to the work of the surgeon. It is useless to add words of proof after what we have seen to-night.

*Dr. Fillebrown.*—We do not want to hear them. I should like a fair statement made as to what you think of the physiological demonstration. Did they speak well?

*Dr. Meriam.*—Certainly they did. I think we can all bear testimony to this. I do not think any of these persons would have any great difficulty in conducting any of the ordinary occupations of life; they are men who have been restored; men that otherwise would have been incapacitated have been restored by this operation so that they can do the ordinary work of life as they could not have done it without this operation.

*Dr. Werner.*—What appears to me worthy of consideration is the changed anatomical condition, the comfort that such must be to the patient, the cleanliness resulting from it. They are certainly in a more normal condition. How much better they speak we cannot tell, but they certainly speak pretty well. How badly they spoke before we can only guess. I am satisfied myself that they are improved. Their anatomical improved condition, their appearance, the satisfaction it must be to them all, I think is a very creditable thing for dentistry to have developed, and considerable credit belongs to the operator, Dr. Fillebrown.

*Dr. Baker.*—Mr. President and gentlemen, when the subject of cleft palate is under discussion, it is generally expected that I will have something to say; also that it has been said that I was prejudiced in regard to the operation. The latter is incorrect. On the other hand, whenever I can see any improvement in the speech after an operation has been performed, I am only too glad to recognize it. To my personal knowledge Dr. Fillebrown has given more or less attention to this subject of cleft palate for the past twenty years. (Dr. Fillebrown: "Halve it.") I go back farther than the period of your surgery, to the time that you first made obturators. To-night is the first time during the entire period that he has given any demonstration that convinces me of improved speech, and that I confine to but one of the cases that he has presented.

In the first case shown, the operation having been performed when the patient was quite young, there was a marked improvement in speech, and he was justified in operating; and I congratulate him upon his success. If all cases were equally favorable, I would never recommend an obturator; but this is one case in five hundred, and of other cases that he has shown I cannot say so much in their favor. My opinion, based on twenty-five years' experience, is that if there is any improvement at all, it is entirely due to vocal training.

Where I criticise Dr. Fillebrown is that he does not discriminate between the different cases, but apparently operates upon cases where there is no more possible chance for improvement in speech than there would be to cut their tongues out. I repeat that I do congratulate him upon the success of the first patient shown here to-night. The result is marvellous.

*President Bradley.*—The next paper is to be illustrated with the stereopticon. Dr. Smith is to present to us a paper entitled "A Contribution to Operative Orthodontia," illustrated with a stereopticon.

(For Dr. Smith's paper, see page 553.)

DISCUSSION.

*President Bradley.*—The subject is now open for discussion. We have had presented to us this evening two papers illustrating work that is being done for people in the clinic of Harvard University, or Harvard Dental School, and I am sure it is a very gratifying exhibit of what is being done in those departments. We shall be very glad to have the papers discussed on their merits, or in any way that you wish.

*Dr. Werner.*—I think dentistry is well advanced over what it was fifty years ago, when we have two subjects like these brought before us in one evening, the outcome of systematized, scientific advancement; and as dentists, in all modesty, we should be proud of our profession and the work going on in our ranks. And when we hear of millions being given for education, I think we, in modesty, can expect or hope that liberal sums should be given to our dental schools. Certainly we are doing good work for humanity. We are curing deformities, we are correcting palates, and we are saving teeth. And if we claim to-day that we are a specialty of medicine, we only need to back up that statement by showing such results.

The predominating thing in my mind is the satisfactory results of modern or scientific dentistry in comparison with twenty-five or thirty years ago. We can be proud of our profession; we ought to love it; and ought to have in our work that high aim that enthusiasm, skill, and science produce. It is the high aim we need, for not failure is crime, but low aim. I think the things we have seen to-night are the results of high aim, faithful work, and scientific study and progress.

May I make this addition, that we could hardly expect a changed condition in a patient of, say, twenty-two years of age or more, in the ramus or in the glenoid cavity, but that we would get results more or less from the tipping of the teeth; but whether or not a change does take place at an earlier age, as in the case spoken of,—that of Dr. Baker's in his own son,—I would like to hear the opinion of the members whether a change takes place in the ramus, in the glenoid cavity, or is it the tipping of the teeth only?

*Dr. Baker.*—Mr. President, if you do not wish me to talk, you must not bring up such interesting subjects for discussion. Before discussing this paper I want to fully understand a sentence that Dr. Smith had in his paper. If I understood him correctly, he made the statement that he believed that it was the teeth tipping, rather than the jaws changing. I want to be clear on that point. It is my opinion, and I have not the slightest doubt about it, that the change is mostly in the jaw. This is something that we wish to prove. It occurred to me, when Dr. Smith was throwing his illustrations upon the screen, that if we could get a little closer together in our views we could better solve this point in view. Before I go any farther, I wish to congratulate Dr. Smith upon bringing these most interesting cases before the society. They certainly have been extremely interesting to me. As I understood it, they were not all completed cases.

I do want to criticise a little: it may be my nature. I think if he had gone a little farther, he would have got better occlusion. Occlusion is vital to the success in regulating, and it is impossible to get teeth to stay in place without it. I have become more and more convinced on this point the past few years, as you know I see a great many of these cases as dentists bring them to me. I have seen many cases where they have attempted to regulate the teeth and simply bring the upper and lower teeth into line, regardless of correct occlusion, resulting in little benefit to the patient.

Another point I wish to speak of, which Dr. Smith did not allude to in his paper, is extracting. I am firmly convinced that it is one of the worst things which can be done in the attempt to regulate teeth. There are very few and rare cases where it is judicious to extract. You cannot get the right relation of the jaws to each other, and it is impossible to do so. You may get fair results, in some cases, but very seldom.

There is one case which Dr. Smith has presented, which he stated was the first case on record that was ever reduced without a chin appliance, where he used the Angle expansion band and the retraction rubber bands. I believe the retraction rubber bands were first introduced by myself, but at that time I had never used them on a protruding lower jaw, but only on the upper jaw. As far as I know, Dr. Smith was the first one to apply them to a protruding lower jaw, which was undoubtedly the first case on record; but I will tell you, gentlemen, I hope to bring the case before the society very soon. When I commenced the case the lower front teeth were out so far one could place their fingers between the upper and lower teeth, and to-day the lower ones are comparatively straight, and the chin has reduced in proportion more than the teeth. And I hope in this case to convince Dr. Smith and all others that the jaw moved rather than the teeth tipped. For the above case I have made a harness, as you might call it, or head-gear, which determines this point definitely. I do not wish to say too much about it now, but prefer to show it to you later. I trust to get the case completed so as to show it at the April meeting, and I anticipate you will be converted, as I said before.

I want to speak a little out of school. That case of a protruding chin I have seen before. As Dr. Smith has said, my son had charge of it under his instructions. The student that was doing the work was over-anxious to get good results too rapidly, so he ground the lower teeth back a little. (Dr. Smith: "The young man was not to blame; he ground the teeth because I told him to.") If he only had been patient, I think that the teeth would have settled themselves into proper occlusion, and the jaws would have closed just as well, and saved the grinding. I do not wish to convey the idea that he spoiled the teeth or injured them, by any means, but I do think it is better not to grind teeth when it can be averted.

*Dr. Meriam.*—Looking at Dr. Smith's appliance, I can see that it has its force beginning and ending on the teeth. But that it has affected the glenoid cavity is not brought out by anything that Dr. Smith has shown to-night. But a chin-piece applied to the head, drawing the jaw into the glenoid cavity, is another apliance and opens up another possibility.

But this thing can be very easily settled. There can be a measure put upon the face, following the lines of the jaw, a flat steel, with a cap for a measure, and then passing by the glenoid cavity, an X-ray taken before the operation is begun. That will show the appliance in place, the position of the glenoid cavity. The operations can then be carried out and the case compared with an X-ray taken when the thing is done. It can, I suppose, be accurately studied in that way and the question determined.

Dr. Smith speaks of the case of twenty-one. I have a similar case. These cases are interesting because they go so far beyond what we hear. A mother said to me that her boy was very ill (he was then nearly twenty), and, tossing on his bed, he said, "I might as well die now, anyway; I have no chin.' Said she, "I never knew you felt that way, Arthur." "Do you suppose I ever forget it?" he said. When she told me, I began on the boy, and I carried the case on with results that were very successful, and that boy gained his chin, at least, as the other teeth were drawn in. Instead of being any longer conscious of himself, he lost all that consciousness, and what went far beyond any mechanical work I had done was the satisfaction given to that boy.

Dr. Smith speaks of the age of the patient when this was begun. I think there is a loss, of course, in the building of new tissue in the security of those teeth afterwards at that age. But there is a great gain in the co-operation that we get from the patient who wishes it done, in their intelligent keeping of appointments, in bearing pain, and that sort of thing, which we may not expect from younger patients.

Here is this evidence presented to-night of what the poor may have done for them in our modern civilization. This is a record that is of credit to our civilization, that children and people can go to an institution and have such intelligent work done with such expert supervision, and these results reached. I do not know of anything of recent years that has added so much. Of course, all through years past we have made the blind to see and the dumb to speak, and all of that, and that was in its day; and now the thing has come into dentistry, and we are doing our part, and I think the exhibition to-night shows that we do it well.

*Dr. Baker.*—There is one point I omitted where Dr. Smith spoke of tipping the anchor teeth. This model I have in my hand with the appliance on it brought it to my mind. The tipping of anchor teeth can be prevented by having the tubes on the anchor band of considerable length. In so doing, what moving there is of the teeth has to be done bodily in the jaw. This necessitates a very slow process, if not an impossible one.

*Dr. Smith.*—There is no need of tipping them.

*Dr. Baker.*—There is no necessity of tipping them more than one one-hundredth of an inch.

*Dr. Meriam.*—That applies to which teeth?

*Dr. Baker.*—Any teeth that you use for anchorage. With a short tube on the band, as they have very little leverage, they will tip; by having a long tube, it absolutely prevents it.

*Dr. Werner.*—Is it not also modified by the position of the tube, whether it is anterior or posterior?

*Dr. Baker.*—I do not think that has any effect as to the tipping.

*Dr. Wilson.*—If I can put in just one word here, I do not think Dr. Baker has explained that fully. For instance, if you have a six-year-old molar tooth, and ligate the front teeth, then put on your maxillary band, you will have a tipping molar; there is no question about that at all. You must ligate your bicuspids next to the molar with the tube; otherwise they will surely tip.

*Dr. Werner.*—One thing should be stated,—namely, that the occlusion of the teeth is a very essential factor in regard to their being retained. We must not be deluded into the idea that we can regulate teeth without occlusion, and retain them. Non-occluded teeth will change again, while the occluded teeth will be kept in place.

*Dr. Wilson.*—The question as to whether there is any change in the glenoid cavity is an extremely interesting one. Personally I do not think there is any change. I think the change is simply in the teeth and in the bone. But it is a very interesting question, and the suggestion that Dr. Meriam made about the X-ray is a very good one.

*Dr. Werner.*—Would not you modify that, Dr. Wilson? Would not you say in a young case the probabilities are that there would be a change in the ramus, beginning from the angle here?

*Dr. Wilson.*—There might be a change in the ramus, but in the glenoid cavity, no. There is a constant change going on in the ramus as one gets older. During childhood the ramus is almost flat, then there is an angle, and in old age it is flat again.

*Dr. Fillebrown.*—Mr. President, I wish to express my appreciation of the completeness as well as the excellence of Dr. Smith's paper, and in looking at these models which have been passed around I take into consideration the criticism that has been made in regard to the occlusion. As an offset to that, I ask any man what fault there is to be found with the occlusion as it appears in the completed cases? I say it is as good as could reasonably be expected by any one in any case under treatment. I do not see why it is not in every way satisfactory. The paper and illustrations are so complete that those of us who at least know no more of the work than the gentleman who presented the paper certainly cannot add much to it.

I would also like to speak of the remarks by Dr. Werner, which hit the nail on the head, as to how to retain the teeth in place. I have one case in hand now that has given me considerable trouble, and another one that I have got an appointment to apply a retaining appliance to, and that is a case of lateral incisors where one is within the arch. There is no occlusion that will hold the tooth in place in either case. I put a band on one, with a brace on the other teeth, and held it there for a year, and after taking it off the teeth receded a good deal.

The one that I have an appointment to complete is a case of a left lateral superior incisor, and I have just got it out in place. The arch is regular and the teeth close together, and I moved it out once, but by the patient's neglect during his absence it got back again. I have not got it into place again. The plan I am going to follow there is that in the cuspid and central there is incipient decay, and I am going to prepare those cavities from the lingual aspect, and put in some fillings and make them prominent enough to form bearings for the teeth and hold them. I tried this some years ago with perfect success.

*Dr. Andrews.*—I have a patient who came to me within a week. I tried to pass a ligature between the cuspid and the lateral; something stopped it, and on examination I found a small gold screw that I had put in twelve years ago to retain the lateral in place, and had forgotten all about it. There was no decay about it.

*Dr. Fillebrown.*—I think I would not hesitate to cut into a sound tooth and make a filling just for that purpose.

*Dr. Baker.*—I have discovered something else which I want to congratulate Dr. Smith on, if he deserves the credit. I do not suppose any one else noticed this hinge. It goes together very easily, but you cannot take it apart unless you tip it 'way over.

*Dr. Smith.*—I do not deserve the credit of that, Mr. President. This hinge is a very clever invention of one of our students, and requires considerable skill in bending the wires.

Adjourned.

CHARLES H. TAFT, D.M.D.,
*Editor American Academy of Dental Science.*

